# Three-Dimensional Porous Graphene Supported MoS_2_ Nanoflower Prepared by a Facile Solvothermal Method with Excellent Rate Performance and Sodium-Ion Storage

**DOI:** 10.3390/polym12092134

**Published:** 2020-09-18

**Authors:** Li Zeng, Liping Zhang, Xingang Liu, Chuhong Zhang

**Affiliations:** 1 State Key Laboratory of Polymer Materials Engineering, Sichuan University, Chengdu 610065, China; zengli_0718@foxmail.com (L.Z.); li_ping_zhang@foxmail.com (L.Z.); 2 Polymer Research Institute, Sichuan University, Chengdu 610065, China

**Keywords:** MoS_2_ nanoflower, three-dimensional porous graphene, sodium-ion batteries, solvothermal

## Abstract

Sodium-ion batteries (SIBs), as a supplement of lithium-ion batteries (LIBs), are attracting intensive research interest due to their low cost and abundance. Molybdenum disulfide (MoS_2_) is regarded as a suitable candidates for SIBs electrode materials, which suffer from prominent volume expansion and poor conductivity. In this study, three-dimensional porous graphene composites loaded with MoS_2_ were prepared via a facile two-step method. The MoS_2_ nanoflower particles were uniformly dispersed within the layered graphene matrix, and a three-dimensional porous graphene supported MoS_2_ nanoflower battery (MoS_2_/3DG) was demonstrated to have superior performance to that of the pristine pure MoS_2_ nanoflower battery. At a current density of 100 mA/g, the MoS_2_/3DG delivered a reversible capacity of 420 mAh/g. What is more, it yielded a reversible specific capacity of 310 mAh/g at 2 A/g, showing an excellent rate of 73.8%. The excellent performance of the novel MoS_2_/3DG composite are attributed to the promoted infiltration of electrolytes and the hindered volume expansion for the porous structure, good conductivity, and robust mechanical properties of graphene.

## 1. Introduction

As rising stars proceding lithium-ion batteries (LIBs), sodium-ion batteries (SIBs) have the advantages of being low in cost, rich in resources, and environmentally-friendly, which play an increasingly important role in the development of power supply, portable electronic equipment, and national defense technology [1,2,3,4]. In recent years, researchers are committed to developing electrode materials with high specific capacity, excellent cycle stability, and safety properties [5,6]. Carbon-based materials are the most popular one in batteries, which can be classified into hard carbon materials and soft carbon materials [7,8]. Hard carbon is usually obtained by pyrolysis of polymer and can provide high specific capacity; however, the relatively high potential of the electrode may cause large irreversible specific capacity during the first charge and discharge process [9,10]. Carbon atoms are highly graphitized and, arranged in order of soft carbon materials, graphite is the most commonly-used soft carbon material with a low discharge voltage platform of ~0.2 V for the LIBs [11]. LiC_6_ compounds are formed when lithium ions intercalate into graphite layers, which can provide a specific capacity of 372 mAh/g [12]. However, graphite is not available in SIBs, for the sodium ions have a large ion radius (0.102 nm vs. Li 0.076 nm), which make it difficult to complete the normal insertion and desorption in the small channels of graphite anode [13,14]. Therefore, it is important to find the anode materials which match well with the radius of sodium ions and have excellent electrochemical performance. In recent years, MoS_2_, as one of the transition metal sulfide has attracted much attention due to its unique two-dimensional layered graphene-like structure, high theoretical specific capacity, and being environmentally friendly [15,16,17]. The two sides of Mo atoms are connected with S atoms through a covalent bond to form a S–Mo–S sandwich structure, while the adjacent S atoms are connected by van der Waals force, and the large layer spacing (0.615 nm vs. graphite 0.335 nm) is suitable for the intercalation and desorption of lithium- and sodium ions [18,19]. Although MoS_2_ has many advantages as an electrode material, it similarly suffers from a large volume expansion coefficient and its volume expansion rate can reach ~300% [20]. At the same time, the stress produced in the charge and discharge process leads to the pulverization of the MoS_2_ electrode and the crystal structure is damaged. Moreover, the conductivity of MoS_2_ material is poor, which severely limits the application at a high current density [21,22,23,24]. In practical researches, MoS_2_ are usually compounded with different dimensions of carbon materials in order to solve these problems. For example, Choi [25] prepared polystyrene nanospheres with a particle size of 100 nm by high temperature spray pyrolysis, and the obtained three-dimensional porous MoS_2_/graphene nanocomposites delivered a discharge capacity and charge specific capacity of 797 and 573 mAh/g, respectively. Most of the preparation operations of the MoS_2_/carbon composites are complex, such as the use of special high-temperature pyrolysis devices and the removal of templates [26,27].

**Figure 1:**
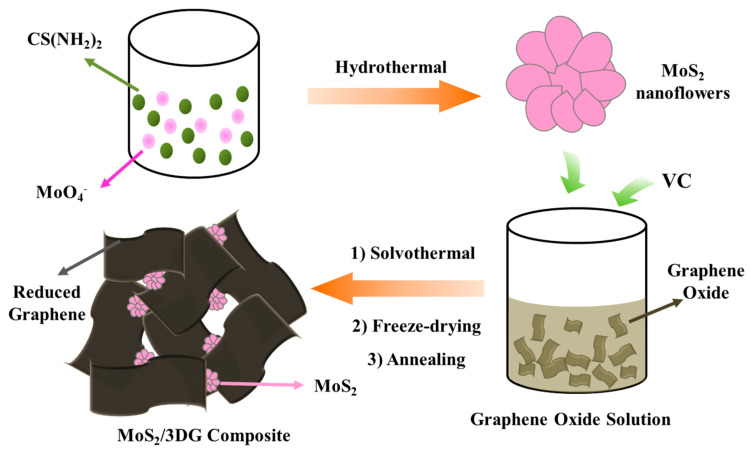
Schematic illustration for the preparation of molybdenum disulfide (MoS_2_)/3DG composite.

Herein, we report a simple two-step method to produce the three-dimensional porous graphene supported MoS_2_ nanoflower composites by a mild solvothermal method (Figure 1). The porous structure is conducive to the full infiltration of electrolyte, which can improve the transmission rate of sodium ions in the electrode. Moreover, the good flexibility of graphene can help to alleviate the volume expansion of MoS_2_ during charge and discharge process and improve the poor conductivity of MoS_2_.

## 2. Materials and Methods

### 2.1. The Preparation of the MoS_2_ Nanoflower Particles

1.07 g ammonium molybdate (NH_4_)_6_Mo_7_O_24_.4H_2_O (Shanghai Aladdin Co., Ltd, Shanghai, China, ≥99.9%) and 0.93 g thiourea CS(NH_2_)_2_ (Shanghai Aladdin Co., Ltd, Shanghai, China, ≥99.9%) were dissolved in 70 mL deionized water and stirred for 1 h, then the mixed solution was transferred to 100 mL hydrothermal reactor and reacted at 200 °C for 24 h. Then, the product was filtered in vacuum and washed with deionized water and absolute ethanol for several times, and then placed in a vacuum oven and dry for 12 h to obtain the MoS_2_ nanoflower particles.

### 2.2. The Preparation of MoS_2_/3DG Composites

The graphite oxide powder (GO, Changzhou Sixth Element Co., Ltd., Changzhou, China) was dispersed in deionized water (concentration of 4 mg/mL), and the graphene oxide suspension was obtained by ultrasonic peeling for 1 h. Then 20 mL of the graphene oxide suspension was transferred into the reactor flask with 160 mg of pre-prepared MoS_2_ nanoflower particles, which were added to the flask during the ultrasonic treatment process. Three hundred and twenty milligrams of vitamin C (Shanghai Aladdin Co., Ltd, China, ≥99.7%) was added for another 10 min ultrasonic treatment, then the sealed reaction flask was place in the mild solvothermal of 80 °C for 8 h to obtain the monolithic hydrogel. The hydrogel was dialyzed with deionized water for 2 days, then freeze-dried to obtain the MoS_2_/3DG composite, placed in a tube furnace under the Ar atmosphere, and thermal treated at 400 °C for 2 h.

### 2.3. Characterizations

The XRD test was carried out on a diffractometer manufactured of Hitachi Company (Tokyo, Japan) with Cu-K α as the radiation source, the scanning speed was 10°/min with a step of 0.01°, the scan range is 5° to 80°. The composition of MoS_2_/3DG was analyzed using the Raman tester (LabRAM HR, HORIBA, Kyoto, Japan). The X-ray photoelectron spectroscopy (XPS) was tested on Nexsa of UK. A thermogravimetric analyzer (TG209F1, Netzsch, Selb, Germany) was used to character the composition ratio of MoS_2_ and graphene in the MoS_2_/3DG composite. The micro-morphology of samples was characterized by the Quanta 200F scanning electron microscope (SEM) and Tecnai G2 transmission electron microscope (TEM) of Philips-FEI company (Hillsboro, OR, USA). The MoS_2_/3DG composite powder, carbon black (conductive agent) and PVDF (binder) were mixed with a mass ratio of 8:1:1, then painted on a pre-treated Cu foil current collector and dried in a vacuum oven at 100 °C for 12 h. The electrode sheets were assembled into the CR2032 button cell in the glove box. The sodium sheet (Afaisa Chemical Co., Ltd, China) was used as the counter electrode, the glass fiber (GF/D, Whatman company, Maidstone, UK) porous membrane was used as the battery separator, and 1 M sodium perchlorate (NaClO_4_) dissolved the mixture of ethylene carbonate (EC) and dimethyl monocarbonate (DMC) with a volume ratio of 1:1 was used as the electrolyte. Electrochemical impedance spectroscopy (EIS) analysis within a frequency range of 100 kHz to 10 mHz with a voltage amplitude of 5 mV and cyclic voltammetry (CV) at different scan rates were performed through VMP3 (Bio-logic company, Grenoble, France), and the cycle stability and rate performance were characterized through the Wuhan Land battery test system.

## 3. Results and Discussion

### 3.1. Structure and Morphology Analysis of MoS_2_ and MoS_2_/3DG Composites

**Figure 2:**
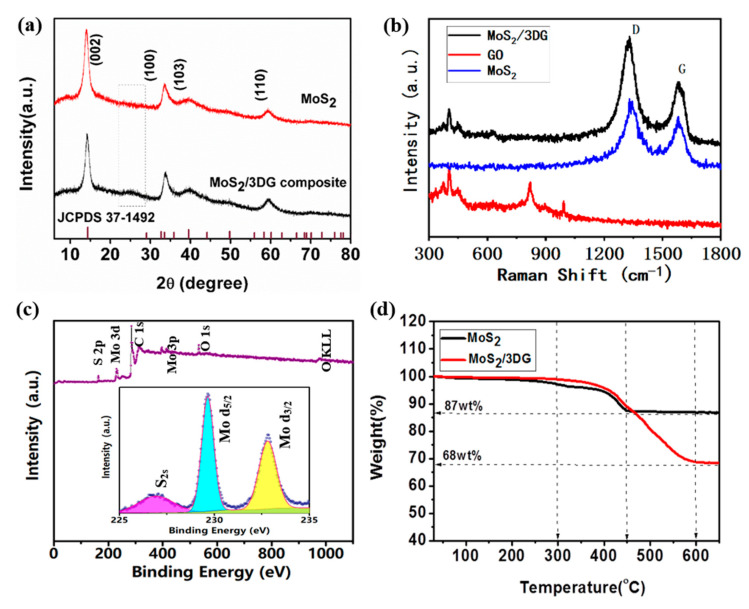
(**a**) XRD and (**b**) Raman of MoS_2_ and MoS_2_/3DG composite, (**c**) XPS curves of MoS_2_/3DG composite and (**d**) TG curves of MoS_2_ and MoS_2_/3DG composite.

The crystalline properties of pure MoS_2_ nanoflower particle and MoS_2_/3DG composite were studied by XRD. As shown in Figure 2a, the diffraction peaks of pure MoS_2_ almost focus on 2θ = 14.2° (002), 33.6° (100), 39.8° (103), and 59.3° (110), corresponding to the hexagonal crystal structure (JCPDS 37-1492) [18]. There are no obvious new diffraction peaks, indicating that a high purity of MoS_2_ particles has been obtained by hydrothermal reaction in the first step. Meanwhile, the diffraction peak positions of the MoS_2_/3DG composite corresponds well with pure MoS_2_ particles, indicating that the crystalline structure of MoS_2_/3DG was not changed by three-dimensional graphene. It is worth noting that a less obvious peak at 25° can be observed, which is attributed to the reduced graphene oxide, for the initial graphene oxide was finally transferred to reduced graphene oxide during the solvothermal and thermal reduction treatment of the second step. The Raman spectra of pure MoS_2_ nanoflower particle, and MoS_2_/3DG composites are shown in Figure 2b, three peaks of pure MoS_2_ can be observed in the low wavenumber band (300–500 cm^−1^), which are attributed to the vibration of the atoms in the hexagonal MoS_2_ [28]. There are two new peaks in the high wavenumber band (1330 and 1580 cm^−1^) of MoS_2_/3DG composites, representing the D-band and G-band of graphene, respectively [29]. The XPS result is shown in Figure 2c, which reveals the presence of the Mo, S, C, and O elements in MoS_2_/3DG; meanwhile, the inset high-resolution XPS spectrum reveals two strong peaks at around 232.8 and 229.6 eV, which can be attributed to the Mo3d_3/2_ and Mo 3d_5/2_ orbitals of Mo^4+^ of the MoS_2_, respectively, while the peak appearing at 226.9 eV is attributed to the S 2s orbital [30,31]. Figure 2d shows the TG curves of pure MoS_2_ and MoS_2_/3DG composites in air, the mass loss of pure MoS_2_ is due to the evaporation of water molecules during the temperature rise from 25 to 300 °C, the mass loss at 300 to 450 °C is due to the conversion of MoS_2_ to MoO_3_ [19], and the residual mass fraction is 87 wt % at 650 °C. Meanwhile, the mass loss of MoS_2_/3DG composites from room temperature to 300 °C is also due to the evaporation of water molecules and the decomposition of residual oxygen-containing functional groups in graphene. The mass loss of MoS_2_/3DG composites at 300 to 600 °C is due to the decomposition of graphene and the conversion of MoS_2_ to MoO_3_, the residual mass fraction was 68 wt % at 650 °C. Hence, the mass of graphene and MoS_2_ in MoS_2_/3DG composites are 22 and 78 wt %, respectively.

**Figure 3:**
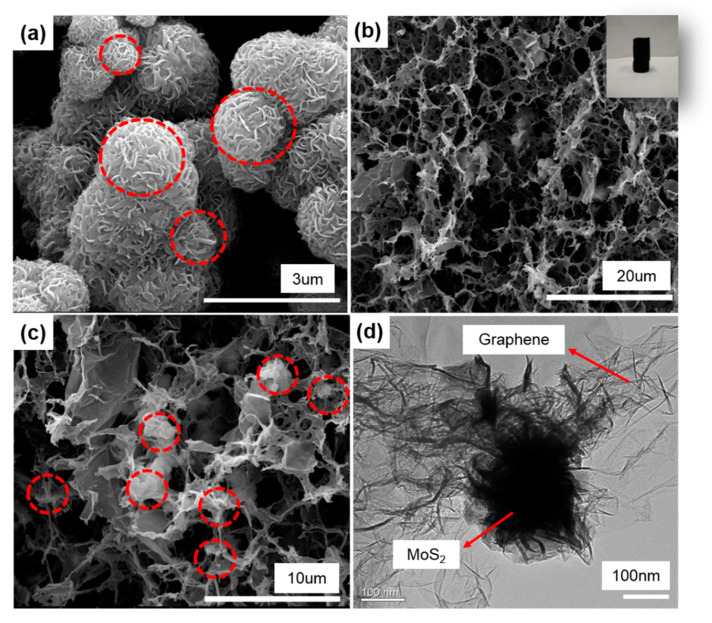
The morphology characterization of pristine MoS_2_ and MoS_2_/3DG composite. (**a**) SEM image of pure MoS_2_ nanoflower particles; (**b**,**c**) SEM images of MoS_2_/3DG composite; (**d**) TEM image of MoS_2_/3DG composite.

**Figure 4:**
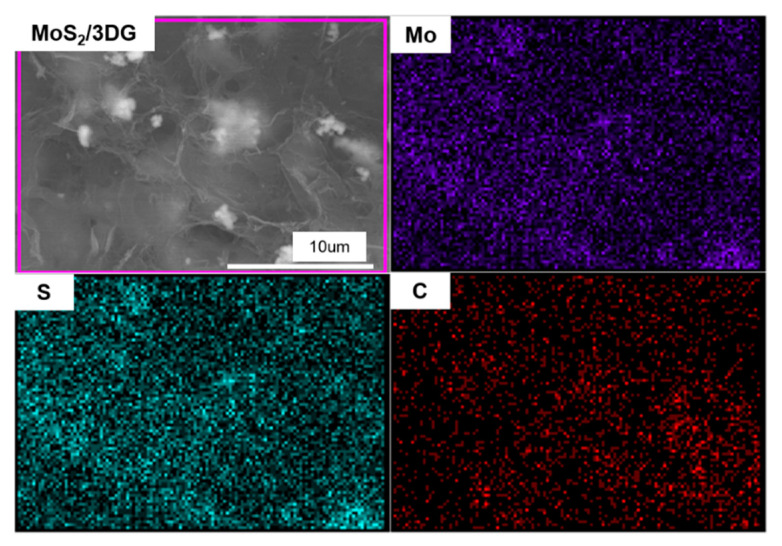
EDS dot-mapping images of Mo, S, and C elements of MoS_2_/3DG composite.

Figure 3a shows the morphology of pure MoS_2_ nanoflower particles with the pure MoS_2_ nanoflower particles composed of stacked nanosheets and closely clustered together. A columnar aerogel of the MoS_2_/3DG composite is shown in the inset of Figure 3b, and the micro morphology is a three-dimensional porous structure. It is noteworthy that the MoS_2_ nanoflower particles are dispersed in the graphene sheet as shown in Figure 3c, the three-dimensional porous structure formed by graphene sheets through mild solvothermal reaction can effectively disperse the MoS_2_ nanoflower particles and reduce the agglomeration. Figure 3d shows the TEM characterization of MoS_2_/3DG composite, graphene sheets are tightly wrapped on the surface of MoS_2_ nanoflower particles, which is benefit to improve the conductivity of the MoS_2_-based composite and alleviate the volume expansion effect of MoS_2_ material during the charge and discharge process. At the same time, the three-dimensional porous structure of the composite will be conducive to the penetration of electrolyte, providing the path for sodium ions and electrons transmission. The further analysis of EDS dot-mapping test reveals the well distribution of Mo, S, and C elements in the MoS_2_/3DG composite as shown in Figure 4.

### 3.2. Electrochemical Analysis of MoS_2_ and MoS_2_/3DG Composites

**Figure 5:**
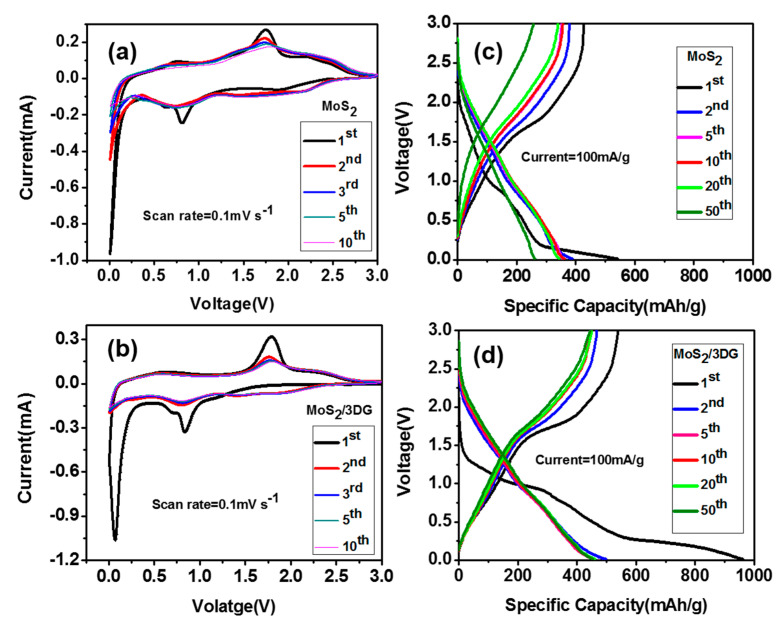
Cyclic voltammograms curves of (**a**) pure MoS_2_ and (**c**) MoS_2_/3DG electrodes; galvanostatic charge-discharge profiles of (**b**) pure MoS_2_ and (**d**) MoS_2_/3DG composite.

The cyclic voltammetry (CV) curves and galvanostatic charge and discharge curves were further tested at the voltage range from 3.0 to 0.01 V vs. Na/Na^+^. As shown in Figure 5a, three obvious reduction peaks of 0.8, 0.65, and 0.01 V and three oxidation peaks of 2.2, 1.7, and 0.75 V appear at the CV curves for the first cycle. From the second cycle, the reduction peak at 0.8 V disappears, and two redox peaks appear at 0.75 V/0.65 V and 1.7 V/1.5 V. The reduction peak at 1.5 V is attributed to the intercalation of Na^+^ into MoS_2_ (MoS_2_ + xNa^+^ + xe^−^ = Na_x_MoS_2_ (x < 2)), while the reduction peak at 0.65 V is attributed to the formation of Mo and Na_2_S (Na_x_MoS_2_ + (4 − x)Na^+^ + (4 − x)e^−^ = 2Na_2_S + Mo) [32]. Figure 5c shows the CV curves of MoS_2_/3DG composite, the peak positions of the CV curves are similar to that of pure MoS_2_, and the coincidence of CV curves at different scan rates are better than that of pure MoS_2_, indicating the good cyclic stability of MoS_2_/3DG composite. Figure 5b,d show the galvanostatic charge and discharge curves with a current density of 100 mA/g. The results show that the discharge platforms of pure MoS_2_ and MoS_2_/3DG composites are about 0.8 V and below 0.5 V and the charging platforms are about 1.7 V, which consistent well with CV results. The MoS_2_/3DG delivers a discharge capacity of 961 mAh/g and a charge capacity of 538 mAh/g for the first cycle, while the discharge and charge capacity of pure MoS_2_ are 541 mAh/g and 428 mAh/g, respectively. After 50 cycles, the specific discharge and charge capacity of MoS_2_/3DG composite are 455 and 444 mAh/g, while pure MoS_2_ deliver the specific discharge and charge capacity of 262 mAh/g and 258 mAh/g, respectively. The excellent electrochemical performance of MoS_2_/3DG composite can be attributed to its unique microstructure, as the graphene sheets lap together to construct a three-dimensional porous conductive network structure, and MoS_2_ nanoflower particles are well-dispersed in graphene sheet layers. What is more, the excellent mechanical properties of graphene can effectively alleviate the volume expansion effect of MoS_2_ in the process of charge and discharge, hence showing much better cycling performance.

**Figure 6:**
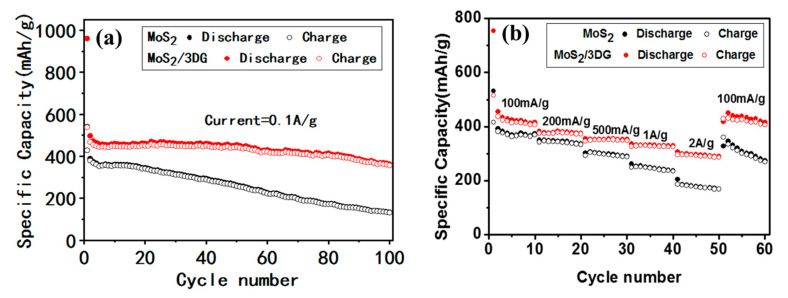
Cycling performance of pristine MoS_2_ and MoS_2_/3DG composite (**a**); rate performance of pristine MoS_2_ and MoS_2_/3DG composite (**b**).

The long-term cycling performance of MoS_2_/3DG and pure MoS_2_ electrodes with a current density of 100 mA/g was evident in Figure 6a. It can be seen that the specific capacity of pure MoS_2_ electrode decreases continuously after 20 cycles, with the discharge specific capacity decreases from 541 to 133 mAh/g within 100 cycles. Meanwhile, the specific discharge capacity of MoS_2_/3DG keeps at 420 and 363 mAh/g after 10 cycles and 100 cycles. Figure 6b shows the rate performance of MoS_2_/3DG and pure MoS_2_ electrodes at different current densities. For the MoS_2_/3DG composite electrode, the revisable specific capacity gently decreases from about 420 to 380 mAh/g, 360, 340, and 310 mAh/g with the current density gradually changing from 100 to 200 mA/g, 500 mA/g, 1A/g, 2A/g, and the reversible specific capacity of the composite rapidly restoring to 420 mAh/g when the current density is restored to 100 mA/g, showing extremely excellent rate of 73.8%. The reversible specific capacity of pure MoS_2_ is 370, 350, 300, 250, and 200 mAh/g with the current density of 100, 200, 500 mA/g, 1 A/g, and 2 A/g, respectively. The reversible specific capacity of pure MoS_2_ decreases much obviously with the current density returns to 100 mA/g, for the structure of pure MoS_2_ is destroyed and powdered due to the huge volume expansion effect during the charging and discharging process, while the graphene in MoS_2_/3DG composite can effectively relieve the volume tension of MoS_2_ and improve the structure stability.

**Figure 7:**
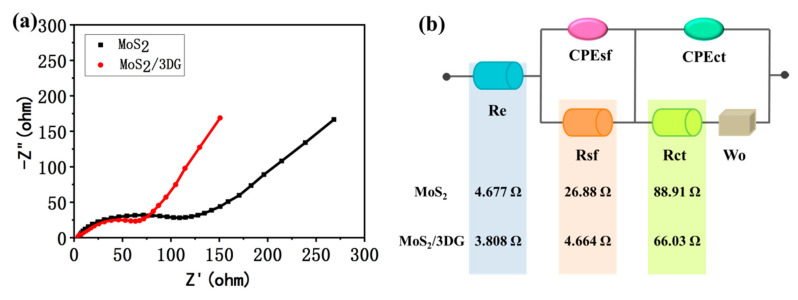
(**a**) The Nyquist plots of pristine MoS_2_ and MoS_2_/3DG composite; (**b**) the equivalent circuits.

The impedance spectra (Figure 7a) of pure MoS_2_ and MoS_2_/3DG composites after 20 cycles of charge and discharge were tested to further reveal their outstanding rate performance. The analysis of impedance spectra is based on the equivalent circuits shown in Figure 7b, where Re represents electrolyte impedance, Rsf represents the interface impedance of SEI film, Rct represents the charge transfer impedance, CPEsf and CPEct represent the SEI film and charge transfer capacitance, respectively, and Zw is the Walberg impedance. The total impedance value (Re + Rsf + Rct) of pure MoS_2_ is 120.5 Ω, while MoS_2_/3DG composite only delivers a total impedance of 74.5 Ω, which is much smaller than that of pure MoS_2_. This indicates that the insertion and desorption of sodium ions in the MoS_2_/3DG composites are much easier, and the redox reactions of charge and discharge can be carried out faster, which can be attributed to the fact that graphene endows the composite with higher conductivity, hence the MoS_2_/3DG composite presents much better electrochemical performance.

## 4. Conclusions

In this work, three-dimensional porous graphene supported MoS_2_ composite MoS_2_/3DG was prepared by mild solvothermal reaction. At the current density of 100 mA/g, the reversible discharge specific capacity of MoS_2_/3DG composite was 420 mAh/g. The electrochemical performance of MoS_2_/3DG composite was greatly improved compared with pure MoS_2_ for its unique three-dimensional porous structure which helped it to permeate the electrolyte, at the same time, the abundant pore structure also helped to shorten the transmission path of sodium ions in the material and improve the transmission rate. In addition, the graphene sheet wrapped on the pure MoS_2_ nanoflower also improved the conductivity. Hence, the capacity of MoS_2_/3DG shows a good rate of 73.8% with the current density increased from 100 mA/g to 2.0 A/g, and the capacity keeps well with original capacity when the current density returned to 100 mA/g. This work provides a new idea for the preparation of high-performance graphene-based electrode materials for sodium ion batteries.
